# Subdivision of IIIC Stage for Endometrioid Carcinoma to Better Predict Prognosis and Treatment Guidance

**DOI:** 10.3389/fonc.2020.01175

**Published:** 2020-07-31

**Authors:** Li Wen, Yanzhen Zhang, Siyuan Chen, Jingjing Wang, Wensheng Hu, Guansheng Zhong

**Affiliations:** ^1^Department of Prenatal Diagnosis and Screening Center, Hangzhou Women's Hospital (Hangzhou Maternity and Child Health Care Hospital), Hangzhou, China; ^2^Department of Breast Surgery, College of Medicine, The First Affiliated Hospital, Zhejiang University, Hangzhou, China; ^3^Department of Obstetrics, Hangzhou Women's Hospital (Hangzhou Maternity and Child Health Care Hospital), Hangzhou, China

**Keywords:** endometrioid cancer, fédération internationale de gynécologie et d'Obstétrique stage, surveillance epidemiology and end results program, nomogram, IIIC stage

## Abstract

**Objective:** The prognostic value of Fédération Internationale de Gynécologie et d'Obstétrique (FIGO) IIIC staging in endometrioid carcinoma patients remains debatable. The current study aimed to compare the prognosis between IIIC1 and IIIC2 patients with endometrioid carcinoma and attempt to conduct a new subdivision.

**Methods:** By using the Surveillance, Epidemiology, and End Results (SEER) database, patients with endometrioid-type endometrial cancer diagnosed from 2004 to 2015 were identified and randomly divided into training and validation sets. We developed a Fine–Gray competing risk model to compare the cancer-specific mortality (CSM). The IIIC subdivision system was built based on the independent prognostic factors. The cumulative incidence curves were compared using Gray's test or log-rank test. Nomogram for predicting 3- or 5-years CSM was constructed and subsequently validated internally and externally.

**Results:** The IIIC subdivision defined by FIGO staging, including IIIC1 and IIIC2, exhibited no association with CSM in multivariate analysis [subdistribution hazard ratio [SHR] = 1.03, 95% CI [0.85–1.26], *P* = 0.760]. The IIIC category was subdivided into three subcategories based on the tumor (T) and nodes (N) stage, including IIICa (T1N1 and T1N2), IIICb (T2N1 and T2N2), and IIICc (T2N1 and T2N2). The prognosis across new IIIC subcategories with CSM remained significant [IIICb vs. IIICa: SHR = 1.53, 95% CI [1.18–1.98], *P* = 0.001; IIICc vs. IIICa: SHR = 2.64, 95% CI [2.13–3.28], *P* < 0.001]. Postoperative adjuvant chemotherapy or radiotherapy alone did not improve survival for patients categorized as IIICa or IIICb, and all IIIC patients benefited most from combination of postoperative chemotherapy and radiotherapy [IIICa: SHR = 0.59, 95% CI [0.43–0.82], *P* = 0.001; IIICb: SHR = 0.66, 95% CI [0.45–0.97], *P* = 0.036; IIICc: SHR = 0.44, 95% CI [0.34–0.58], *P* < 0.001]. A nomogram based on competing risk model was built to predict the long-term survival of IIIC patients, with a concordance index above 0.70 both in training and validation set.

**Conclusion:** There was no prognostic difference between FIGO IIIC1 and IIIC2 patients with endometrioid-type endometrial cancer. A new subdivision of IIIC category facilitates prognosis prediction and treatment modalities. A combination of postoperative chemotherapy and radiotherapy exerted as the optimal choice for endometrioid cancer patients with IIIC stage.

## Introduction

Endometrial carcinoma is the most commonly diagnosed gynecologic malignancy in women, accounting for ~7% of all newly diagnosed cancer and ~4% of cancer-directed deaths in the United States. According to the latest cancer statistical report, an estimated 63,230 new cases of endometrial cancer are expected to be diagnosed in 2018 in the United States nationally, with 11,350 deaths caused by this disease (1). Generally, the majority of patients with endometrial cancer, ~75%, are diagnosed at an early stage because of the early symptom of vaginal bleeding, resulting in overall favorable prognosis with a 5-years overall survival rate of 80 to 85% (2). Nevertheless, a substantial proportion of patients have suffered advanced-stage disease, stage III–IV, which is associated with a higher recurrence rate and dismal clinical outcome (3). It was reported that the 5-years survival rate for stages III and IV endometrial cancer is approximately as low as 45–60% and 15–25%, respectively (4).

Patients with endometrial cancer who suffered from lymphatic metastasis is classified as stage IIIC (5). Pelvic lymph node metastases occur in ~10% of patients with clinical early-stage endometrial cancer and are increasingly found in advanced stage with higher tumor grade and deeper invasion (6, 7). The involvement of retroperitoneal lymph node, either pelvic or para-aortic lymph nodes, results in a worse prognosis (8). It is also well-known that the Fédération Internationale de Gynécologie et d'Obstétrique (FIGO) staging further divided stage IIIC into stage IIIC1 (pelvic lymph node involvement) and IIIC2 (aortic ± pelvic lymph node involvement) in 2009 (9). However, although aortic lymph node involvement suggests further progression of the disease, there has been limited data for the prognostic significance for this IIIC stage revision, especially for endometrioid endometrial carcinoma (EEC), which often have a good prognosis. The tumor (T) stage, which is often applied to assist clinicians in prognosis prediction, makes no sense in staging patients diagnosed as IIIC stage. Furthermore, the possible benefits of multiple treatment modalities, including different types of surgery and adjuvant treatment, may be obscured due to the heterogeneity of those patients just designated as “stage IIIC.” Consequently, given the considerably differential prognosis of patients with stage IIIC endometrioid cancer, it is inappropriate to assign those patients a “catch-all” classification (10, 11). Further optimization of the existing IIIC staging for endometrioid cancer is of clinical importance for accurate prediction of patient's prognosis and personalized treatment modalities.

Therefore, the purpose of our study was to evaluate the prognostic value of the current FIGO IIIC staging in endometrioid cancer and attempt to divide patients into different risk groups, that is, subsets of stage IIIC. In this study, through multivariate competitive risk model, we exploited the data from the Surveillance, Epidemiology, and End Results (SEER) program to stratify endometrioid cancer patients with IIIC stage into different subcategories based on the American Joint Committee on Cancer (AJCC) T and nodes (N) stage. The survival benefit of multiple combinations of treatment modalities both in the total set and the new subsets of stage IIIC patients was studied. Also, a nomogram based on the multivariate competitive risk model was subsequently built for predicting the long-term survival of endometrioid cancer patients with IIIC stage. After that, the nomogram was separately validated in the validation set. We hope that those new subdivisions as well as nomogram of IIIC stage for endometrioid carcinoma may assist clinicians in classifying patients with distinct prognoses and guide appropriate treatment.

## Materials and Methods

### Database and Case Selection

Population-based data were obtained from the recently released SEER database [Incidence—SEER 18 Regs Custom Data [with additional treatment fields], Nov 2018 Sub], which collects information of cancer patients that covers ~28% of the US population (12). The SEER^*^Stat software version 8.3.6 (National Cancer Institute, USA) was utilized to access the data from the SEER database. With the permission from the SEER program office, a total of 4,931 patients with stage IIIC endometrioid-type endometrial cancer (International Classification of Diseases for Oncology, third edition, histologic type/behavior code: 8380/3) who were diagnosed from 2004 to 2015 were extracted from the database. Then, patients with unknown AJCC T stage, unknown radiation information, unknown surgery information, unknown specific IIIC stage, or incomplete follow-up were excluded. Also, only patients with one primary malignancy only were included. Finally, a total of 3,591 eligible endometrioid cancer patients with IIIC stage were included in this study and subsequently divided randomly into the training and validation sets using the basic R function *sample ()*. In detail, 1,795 patients were included in the training set, and 1,796 patients were included in the validation set. The flowchart of case selection can be seen in Supplementary Figure 1.

### Co-variates

The analysis in this study involved variables including demographic characteristics (age at diagnosis, race, and marital status), disease characteristics (histologic grade, AJCC T stage, and AJCC N stage), and treatment characteristics (chemotherapy, radiation, and surgery). The seventh edition of the AJCC tumor–nodes–metastases staging was utilized in this study.

Especially, age at diagnosis, a continuous variable, was transformed into categorical variables (<40, 41–60, 61–80, and ≥80). Vital status record and cause-specific death classification were used to define the main outcomes, including cancer-specific mortality (CSM) and all-cause mortality.

### Statistical Analysis

Descriptive statistics was utilized to summarize the baseline characteristics of endometrioid carcinoma. Clinicopathologic characteristics between the training and validation sets were compared using Pearson chi-square tests. Cumulative incidence curves of CSM and all-cause mortality were compared based on the log-rank test or Gray's test (13). The univariate and multivariate analyses were performed using the Fine–Gray regression model to estimate subdistribution hazard ratios (SHRs) for CSM (14). Meanwhile, the multivariate Cox proportional hazards model using a backward stepwise approach was also constructed to estimate the hazard ratios (HRs) for all-cause mortality. Regarding the classification method of the new subdivision of the IIIC category, the total patients in the training set were firstly divided into six subgroups (T1N1, T1N2, T2N1, T2N2, T3N1, and T3N2) according to the AJCC T and N stage. Then, multiple comparisons among those subgroups for CSM were performed using the multivariate Fine–Gray regression model. Based on the result of the multivariate Fine–Gray regression model, groups with significant differences in survival (*P* < 0.05) were divided into different IIIC subcategories, whereas groups with no significantly different prognosis were merged into one category. For example, there was no significant survival difference between patients with T1N2 and those with T1N1. Then, those two subgroups were merged. On the contrary, patients with T2N1 had significantly poor prognosis compared with patients with T1N1 or T1N2. As a result, those subgroups were classified into different subcategories. Also, cumulative incidence rates for CSM in new IIIC subcategories were estimated and compared according to Gray's test.

Also, Gray's test and a Fine–Gray regression model were conducted to put up multiple comparisons across new IIIC subcategories for survival benefit of different treatment modalities by setting up a different reference. Subsequently, a nomogram was developed according to the multivariate result of the Fine–Gray regression model for predicting the probability of CSM in 3 or 5 years. The concordance index (C-index) was calculated to assess the discrimination of the nomogram, whereas calibration curves were plotted to estimate the consistency between the actual observed outcome and nomogram-predicted probability. Moreover, the nomogram was also validated in the validation set by calculating the C-index and plotting the calibration curves. A bootstrap method with 1,000 resamples was used to validate the nomograms both internally and externally.

Descriptive statistics, Pearson Chi-square test, and the Cox proportional hazards model were performed using SPSS 24.0 (IBM Corp). Gray's test, Fine–Gray regression model, nomogram, and calibration curves were conducted or plotted by using R software version 3.6.0. A two-sided *P* < 0.05 was considered as statistical significance unless otherwise stated. ^*^p < 0.05, ^**^p < 0.01, and ^***^p < 0.001.

## Results

### Baseline Characteristics of Patients in the Datasets

Through rigorous screening, a total of 3,591 eligible endometrioid cancer patients with IIIC stage, who were diagnosed from 2004 to 2015, were included in this study. To perform an external validation, the total patients were randomly divided into training and validation sets. Detailed baseline characteristics of the two sets are outlined in Table S1. In general, there was no significant difference in demographic and clinicopathological characteristics between the two sets.

### Association Between Fédération Internationale de Gynécologie et d'Obstétrique IIIC Stage and Cancer-Specific Mortality

For better understanding, the clinicopathological factors associated with CSM of endometrioid cancer patients with IIIC stage, univariate and multivariate competitive risk analyses were performed (Table 1). Univariate analysis showed that age ≥80 years, black women, not married, higher histologic grade, advanced AJCC T stage, and not receiving treatment were associated with increased CSM. Multivariable analysis showed that older age, marital status, histologic grade, AJCC T stage, and treatment like surgery and chemotherapy remained a significant predictor of CSM (P < 0.05). Interestingly, the IIIC subdivision defined by FIGO staging, including IIIC1 and IIIC2, exhibited no association with CSM both in univariate [SHR = 1.20, 95% CI [0.99–1.44], *P* =0.054] and multivariate analyses [SHR = 1.03, 95% CI [0.85–1.26], *P* = 0.760]. Hence, we further analyzed the prognostic value of FIGO IIIC staging in the validation and total sets (Table 2). Although FIGO IIIC staging significantly associated with CSM in univariate analysis, the multivariate analysis demonstrated that it was no longer an independent prognostic factor both in the training set [SHR = 1.19, 95% CI [0.98–1.44], *P* = 0.069] and total set [SHR = 1.11, 95% CI [0.97–1.28], *P* = 0.130] after adjusting for other clinicopathological co-variants. Moreover, in the subgroup stratified by T stage, there was still no prognostic difference between IIIC1 and IIIC2 subgroups both in univariate and multivariate analyses (*P* ≥ 0.05) (Table 2). Those data, to some extent, were not consistent with the original purpose of clinical staging, that is, to better distinguish the prognosis of disease.

**Table 1:** Univariate and multivariate analyses of cancer-specific mortality in the training set: a competing risk regression model.

		**Univariate analysis**		**Multivariate analysis**	
	***n***	**SHR (95% CI)**	***P*-value**	**SHR (95% CI)**	***P*-value**
**Age, y**					
<40	46	Ref		Ref	
41–60	818	0.99 (0.55–1.79)	0.970	1.15 (0.62–2.12)	0.660
61–80	834	1.62 (0.90–2.91)	0.110	1.89 (1.03–3.47)	0.041
>81	97	3.43 (1.79–6.54)	<0.001	2.90 (1.50–5.63)	0.002
**Race**					
Black	171	Ref		Ref	
White	1,423	0.56 (0.43–0.73)	<0.001	0.75 (0.54–1.03)	0.078
Other	201	0.43 (0.30–0.64)	<0.001	0.67 (0.44–1.03)	0.065
**Marital status**					
Unmarried	875	Ref		Ref	
Married	852	0.67 (0.56–0.80)	<0.001	0.77 (0.64–0.94)	0.009
Unknown	68	0.61 (0.33–1.12)	0.110	0.67 (0.35–1.28)	0.220
**Histologic grade**					
Grade 1	324	Ref		Ref	
Grade 2	600	1.56 (1.12–2.18)	0.008	1.42 (1.02–1.98)	0.040
Grade 3	478	3.38 (2.45–4.67)	<0.001	2.51 (1.79–3.51)	<0.001
Grade 4	71	4.11 (2.61–6.48)	<0.001	3.35 (2.13–5.26)	<0.001
Unknown	322	2.18 (1.51–3.15)	<0.001	2.04 (1.41–2.97)	<0.001
**AJCC T stage**					
T1	910	Ref		Ref	
T2	363	1.71 (1.33–2.18)	<0.001	1.53 (1.18–1.98)	0.001
T3	522	3.19 (2.61–3.91)	<0.001	2.64 (2.13–3.28)	<0.001
**FIGO stage**					
IIIC1	1,190	Ref		Ref	
IIIC2	605	1.20 (0.99–1.44)	0.054	1.03 (0.85–1.26)	0.760
**Surgery**					
No	48	Ref		Ref	
Yes	1,747	0.13 (0.09–0.19)	<0.001	0.17 (0.11–0.26)	<0.001
**Chemotherapy**					
No	556	Ref		Ref	
Yes	1,239	0.63 (0.53–0.75)	<0.001	0.70 (0.57–0.85)	<0.001
**Radiotherapy**					
No	690	Ref		Ref	
Yes	1,105	0.66 (0.56–0.79)	<0.001	0.86 (0.71–1.04)	0.110

*AJCC, American Joint Committee on Cancer; FIGO, Fédération Internationale de Gynécologie et d'Obstétrique; SHR, subdistribution hazard ratio*.

**Table 2:** Association of cancer-specific mortality with FIGO IIIC stage.

	**Training set**			**Validation set**			**Total set**		
	***n***	**SHR (95% CI)**	***P*-value**	***n***	**SHR (95% CI)**	***P*-value**	***n***	**SHR (95% CI)**	***P*-value**
**Part I: univariate analysis**									
Total group	1,795			1,796			3,591		
IIIC1	1,190	Ref		1,226	Ref		2,416	Ref	
IIIC2	605	1.20 (0.99–1.44)	0.054	570	1.39 (1.16–1.67)	<0.001	1,175	1.29 (1.14–1.47)	<0.001
T1 subgroup	910			907			1,817		
IIIC1	636	Ref		651	Ref		1,287	Ref	
IIIC2	274	1.07 (0.76–1.52)	0.700	256	1.15 (0.83–1.59)	0.400	530	1.12 (0.88–1.41)	0.370
T2 subgroup	363			355			718		
IIIC1	257	Ref		253	Ref		510	Ref	
IIIC2	106	1.10 (0.73–1.68)	0.650	102	1.57 (1.04–2.36)	0.03	208	1.31 (0.98–1.76)	0.07
T3 subgroup	522			534			1,056		
IIIC1	297	Ref		322	Ref		619	Ref	
IIIC2	225	1.05 (0.81–1.36)	0.710	212	1.23 (0.95–1.60)	0.11	437	1.14 (0.95–1.36)	0.17
**Part II: multivariate analysis^a^**									
Total group	1,795			1,796			3,591		
IIIC1	1,190	Ref		1,226	Ref		2,416	Ref	
IIIC2	605	1.03 (0.85–1.26)	0.760	570	1.19 (0.98–1.44)	0.069	1,175	0.11 (0.97–1.28)	0.130
T1 subgroup	910			907			1,817		
IIIC1	636	Ref		651	Ref		1,287	Ref	
IIIC2	274	1.09 (0.75–1.56)	0.660	256	1.05 (0.75–1.49)	0.770	530	1.06 (0.83–1.36)	0.630
T2 subgroup	363			355			718		
IIIC1	257	Ref		253	Ref		510	Ref	
IIIC2	106	1.12 (0.72–1.75)	0.600	102	1.54 (1.00–2.37)	0.050	208	1.25 (0.92–1.68)	0.150
T3 subgroup	522			534			1,056		
IIIC1	297	Ref		322	Ref		619	Ref	
IIIC2	225	0.93 (0.71–1.23)	0.610	212	1.20 (0.91–1.58)	0.200	437	1.07 (0.88–1.30)	0.480

*FIGO, Fédération Internationale de Gynécologie et d'Obstétrique; SHR, subdistribution hazard ratio*.

a *The multivariate analysis was adjusted for age, race, marital status, histologic grade, American Joint Committee on Cancer T stage, and treatment modalities (surgery, chemotherapy, and radiation)*.

### Subdivision of IIIC Category and Its Prognostic Significance

**Figure 1:**
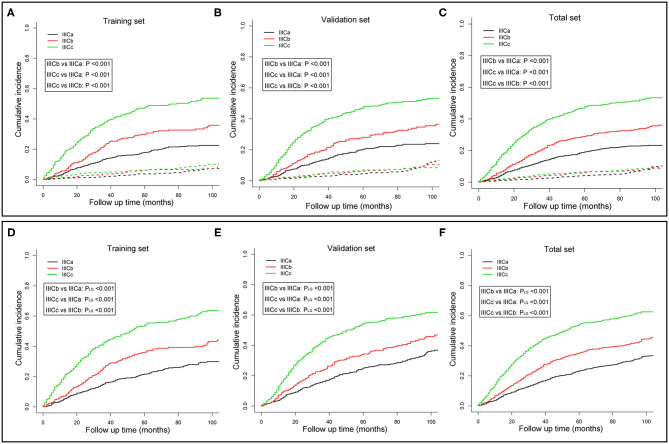
Cumulative incidence curves of cancer-specific mortality **(A–C)** and all-cause mortality **(D–F)** for different IIIC subcategories in the training **(A,D)**, validation **(B,E)**, and whole sets **(C,F)**. *P*-values were compared using Gray's test **(A–C)** or log-rank test **(D–F)**. The solid and dashed lines in **A**–**C** denote cancer-specific death (CSD) and non-cancer specific death (non-CSD). IIICa consists of patients with T1N1 or T1N2; IIICb consists of patients with T2N1 or T2N2; IIICc consists of patients with T3N1 or T3N2.

To better distinguish IIIC stage patients with distinct clinical outcomes, patients in the training set were further divided into six subgroups (T1N1, T1N2, T2N1, T2N2, T3N1, and T3N2) based on the T and N stage. The CSM among those subgroups was subject to multiple comparisons using the univariate and multivariate competing risk models (Table S2). Consequently, based on the independent prognostic factors obtained from multivariate analysis, patients with IIIC stage endometrioid cancer were subdivided into three new categories: IIICa, patients with T1N1 or T1N2; IIICb, patients with T2N1 or T2N2; and IIICc, patients with T3N1 or T3N2. Both the CSM and all-cause mortality rates increased dramatically across the new IIIC subcategories (Figures 1A,D). A similar tendency was also exhibited in the validation (Figures 1B,E) and total sets (Figures 1C,F). In the adjusted competing risk model, the prognosis across the IIIC subcategories with CSM remained significant [IIICb vs. IIICa: SHR = 1.53, 95% CI [1.18–1.98], *P* = 0.001; IIICc vs. IIICa: SHR = 2.64, 95% CI [2.13–3.28], *P* < 0.001] (Table 3 and Table S3). Moreover, the IIIC subcategories were also dramatically associated with increased all-cause mortality [IIICb vs. IIICa: HR = 1.51, 95% CI [1.21–1.90], *P* < 0.001; IIICc vs. IIICa: HR = 2.67, 95% CI [2.21–3.23], *P* < 0.001] (Table 3 and Table S4). Consistently, the prognostic difference across the IIIC subcategories was well validated in the validation and total sets (Table 3 and Tables S3, S4).

**Table 3:** Multivariate analysis of cancer-specific and all-cause mortality^a^.

	**Training set**		**Validation set**		**Whole set**	
	**SHR (95% CI)**	***P*-value**	**SHR (95% CI)**	***P*-value**	**SHR (95% CI)**	***P*-value**
**Part I: Cancer-specific mortality**						
IIICa	Ref		Ref		Ref	
IIICb	1.53 (1.18–1.98)	0.001	1.35 (1.05–1.74)	0.021	1.45 (1.21–1.73)	<0.001
IIICc	2.64 (2.13–3.28)	<0.001	2.45 (1.98–3.03)	<0.001	2.52 (2.17–2.93)	<0.001
	**HR (95% CI)**	***P*****-value**	**HR (95% CI)**	***P*****-value**	**HR (95% CI)**	***P*****-value**
**Part II: All-cause mortality**						
IIICa	Ref		Ref		Ref	
IIICb	1.51 (1.21–1.90)	<0.001	1.33 (1.02–1.58)	0.033	1.41 (1.21–1.65)	<0.001
IIICc	2.67 (2.21–3.23)	<0.001	2.27 (1.88–2.73)	<0.001	2.45 (2.15–2.80)	<0.001

*Part I: competing risk regression model; Part II: Cox proportional hazards model; IIICa: patients with T1N1 or T1N2; IIICb: patients with T2N1 or T2N2; IIICc: patients with T3N1 or T3N2*.

a *Adjusted variables included age, race, marital status, histologic grade, Fédération Internationale de Gynécologie et d'Obstétrique stage, and treatment including surgery, chemotherapy, and radiation*.

### Associations of Treatment Modality and IIIC Subcategories With Survival Outcomes

**Figure 2:**
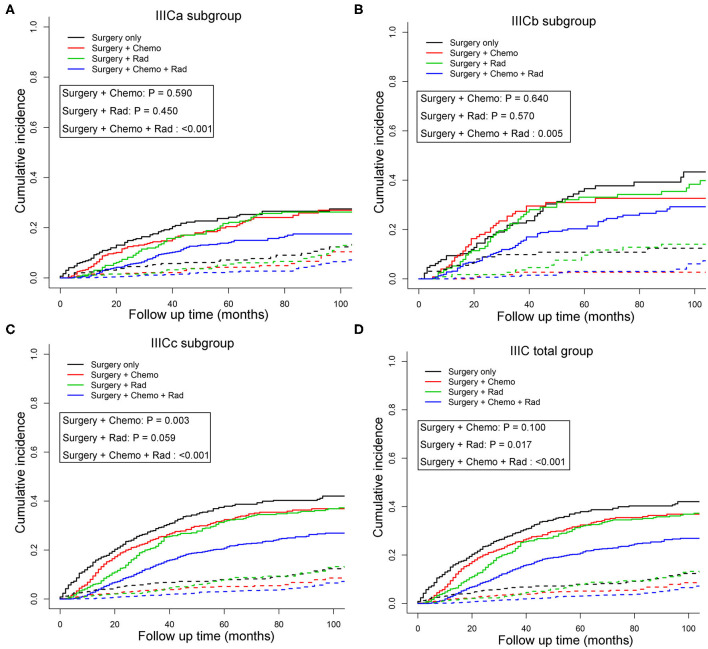
Cumulative incidence curves of cancer-specific mortality stratified by treatment modalities in patients with IIICa **(A)**, IIICb **(B)**, IIICc **(C)**, and IIIC **(D)** in the total set. Patients receiving surgery only were set as the reference and the *P*-values for Gray's test were presented. The solid and dashed lines in **A**–**C** denote cancer-specific death (CSD) and non-cancer specific death (non-CSD). IIICa consists of patients with T1N1 or T1N2; IIICb consists of patients with T2N1 or T2N2; IIICc consists of patients with T3N1 or T3N2.

To better understand the survival benefit of different treatment modalities, the cumulative incidence curves of CSM among various combinations of therapeutic modalities were compared (Figures 2A–D). Although patients who received surgery exhibited the lowest SHR in both sets [training set: SHR = 0.17, 95% CI [0.11–0.26], *P* < 0.001; validation set: SHR = 0.19, 95% CI [0.12–0.28], *P* < 0.001; total set: SHR = 0.20, 95% CI [0.15–0.27], *P* < 0.001] (Table S3), it is our belief that surgery is necessary for patients with stage IIIC in any case because of the great survival benefits. When compared with the monotherapy of surgery alone, the combination treatments, including postoperative radiotherapy or postoperative chemotherapy, and radiotherapy were significantly associated with favorable prognosis in the total IIIC patient group (*P* < 0.05) (Figure 2D). Also, for the subgroup of patients identified as IIICa and IIICb, only combination therapy (postoperative chemotherapy and radiotherapy) showed survival benefit compared with surgery only (*P* < 0.001) (Figures 2A,B). Nevertheless, for patients with IIICc, surgery plus chemotherapy also increased the survival rate compared with surgery only (*P* < 0.01) (Figure 2C). The patient demographics of various treatment modalities are displayed in Table S5.

Subsequently, multivariate competing risk models were utilized to unveil the independent prognostic indicators of combined treatment modalities compared with surgery only (Table 4). Similarly, except for postoperative chemotherapy, IIIC patients could further benefit from radiotherapy [SHR = 0.76, 95% CI [0.62–0.94], *P* = 0.012] or postoperative chemotherapy and radiotherapy [SHR = 0.55, 95% CI [0.46–0.66], *P* < 0.001) when compared with patients who received surgery only. For subgroups with IIICa and IIICb, only those who received the combination of postoperative chemotherapy and radiotherapy had survival benefit in comparison with those who had surgery only [IIICa: SHR = 0.59, 95% CI [0.43–0.82], *P* =0.001; IIICb: SHR = 0.66, 95% CI [0.45–0.97], *P* = 0.036]. However, for subgroups with IIICc, any combination treatment modalities exhibited better than monotherapy with surgery only [surgery + chemotherapy: SHR = 0.68, 95% CI [0.51–0.90], *P* = 0.007; surgery + radiotherapy: SHR = 0.67, 95% CI [0.48–0.94], *P* = 0.020; surgery + chemotherapy + radiotherapy: SHR = 0.44, 95% CI [0.34–0.58], *P* < 0.001]. Importantly, taken all these results into consideration, those adjusted competing risk models demonstrated that combination therapy of postoperative chemotherapy and radiotherapy exerted as the optimal choice for endometrioid cancer patients with IIIC stage (Table 4).

**Table 4:** Association of cancer-specific mortality with treatment modality.

	**Model 1**		**Model 2**		**Model 3**	
	**SHR (95% CI)**	***P*-value**	**SHR (95% CI)**	***P*-value**	**SHR (95% CI)**	***P*-value**
**Part I: univariate analysis**						
**IIIC**						
Surgery only	Ref					
Surgery + Chemo	0.85 (0.71–1.03)	0.100	Ref			
Surgery + Rad	0.78 (0.64–0.96)	0.017	0.91 (0.75–1.11)	0.370	Ref	
Surgery + Chemo + Rad	0.49 (0.41–0.58)	<0.001	0.57 (0.48–0.68)	<0.001	0.63 (0.52–0.76)	<0.001
**IIICa**						
Surgery only	Ref					
Surgery + Chemo	0.91 (0.62–1.27)	0.590	Ref			
Surgery + Rad	0.88 (0.62–1.23)	0.450	1.04 (0.69–1.33)	0.800	Ref	
Surgery + Chemo + Rad	0.53 (0.39–0.73)	<0.001	0.58 (0.43–0.79)	<0.001	0.61 (0.45–0.83)	0.002
**IIICb**						
Surgery only	Ref					
Surgery + Chemo	0.90 (0.58–1.40)	0.640	Ref			
Surgery + Rad	0.89 (0.58–1.35)	0.570	0.99 (0.63–1.55)	0.950	Ref	
Surgery + Chemo + Rad	0.59 (0.41–0.86)	0.005	0.66 (0.44–0.99)	0.044	0.67 (0.45–0.98)	0.039
**IIICc**						
Surgery only	Ref					
Surgery + Chemo	0.66 (0.50–0.87)	0.003	Ref			
Surgery + Rad	0.73 (0.53–1.01)	0.059	1.11 (0.82–1.50)	0.490	Ref	
Surgery + Chemo + Rad	0.37 (0.29–0.49)	<0.001	0.56 (0.44–0.72)	<0.001	0.51 (0.38–0.68)	<0.001
**Part II: multivariate analysis^a^**						
**IIIC**						
Surgery only	Ref					
Surgery + Chemo	0.86 (0.71–1.05)	0.130	Ref			
Surgery + Rad	0.76 (0.62–0.94)	0.012	0.89 (0.72–1.08)	0.250	Ref	
Surgery + Chemo + Rad	0.55 (0.46–0.66)	<0.001	0.64 (0.54–0.76)	<0.001	0.73 (0.60–0.88)	0.001
**IIICa**						
Surgery only	Ref					
Surgery + Chemo	0.93 (0.66–1.31)	0.680	Ref			
Surgery + Rad	0.81 (0.57–1.14)	0.230	0.87 (0.62–1.22)	0.410	Ref	
Surgery + Chemo + Rad	0.59 (0.43–0.82)	0.001	0.64 (0.47–0.86)	0.003	0.73 (0.53–1.02)	0.065
**IIICb**						
Surgery only	Ref					
Surgery + Chemo	0.96 (0.62–1.50)	0.860	Ref			
Surgery + Rad	0.96 (0.62–1.49)	0.860	1.00 (0.63–1.58)	0.990	Ref	
Surgery + Chemo + Rad	0.66 (0.45–0.97)	0.036	0.69 (0.46–1.03)	0.072	0.69 (0.46–1.03)	0.070
**IIICc**						
Surgery only	Ref					
Surgery + Chemo	0.68 (0.51–0.90)	0.007	Ref			
Surgery + Rad	0.67 (0.48–0.94)	0.020	0.99 (0.72–1.36)	0.930	Ref	
Surgery + Chemo + Rad	0.44 (0.34–0.58)	<0.001	0.65 (0.51–0.84)	<0.001	0.66 (0.49–0.91)	0.010

*SHR, subdistribution hazard ratio; Chemo, chemotherapy; Rad, radiotherapy*.

a *Adjusted variables included age, race, marital status, histologic grade, Fédération Internationale de Gynécologie et d'Obstétrique stage*.

### Construction of Nomogram for IIIC Patients and External Validation

**Figure 3:**
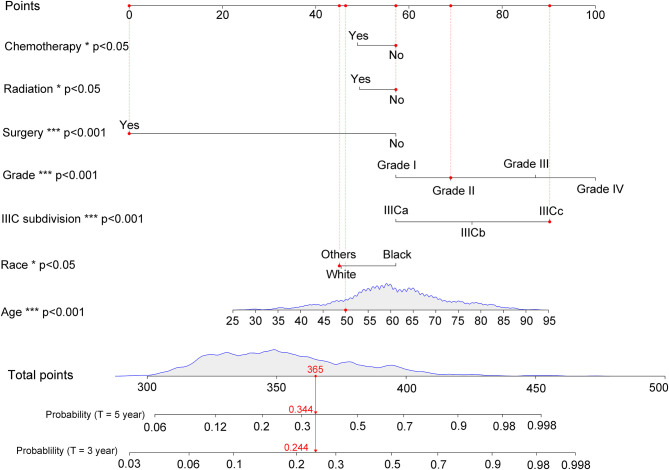
Nomogram for predicting 3- and 5-years cancer-specific mortality with IIIC endometrioid carcinoma. The detailed scores for a randomly selected patient were represented using the red dashed line and font. IIICa consists of patients with T1N1 or T1N2; IIICb consists of patients with T2N1 or T2N2; IIICc consists of patients with T3N1 or T3N2. *p < 0.05, **p < 0.01, and ***p < 0.001.

According to the results of multivariate competing risk analysis, a nomogram was plotted specifically for IIIC patients to predict the 3- and 5-years CSM. The nomogram incorporated seven variables (age, race, histologic grade, new IIIC subdivision, surgery, chemotherapy, and radiotherapy) that were demonstrated to be independent prognostic factors with statistical significance in multivariate analysis (*P* < 0.05). Based on the point scale in the nomogram, a total point could be calculated for each IIIC patient by referring to their different clinicopathologic characteristics, which provided a clear and concise way to predict the long-term CSM. As shown in Figure 3, for instance, randomly selected patients showed a total point of 365 based on its individual clinicopathological features. As a result, the 3- and 5-years CSM for these patients were 24.4 and 34.4%, respectively. Generally speaking, patients with a higher score was deemed to have a worse prognosis. Subsequently, the nomogram was validated internally and externally by calculating the C-index. The C-index was 0.756 (95% CI: 0.733–0.779) in the training set and 0.736 (95% CI: 0.711–0.761) in the validation set, suggesting an acceptable predictive accuracy. Moreover, through plotting the calibration curves, the nomogram showed good consistency between the observed survival probability and the nomogram-predicted survival probability both in the training and validation sets (Supplemental Figure 2).

## Discussion

Endometrial cancer spreads beyond the uterus by metastasizing most often to pelvic nodes and secondarily or less frequently directly to the para-aortic nodes (15). With the recognition that para-aortic lymph nodes involvement likely suggests further progression and connotes a bad prognosis (16), the 2009 revised FIGO staging system further stratified IIIC stage into IIIC1 with pelvic nodes involvement only and IIIC2 with aortic regardless of pelvic nodes metastasis (17). Although the revision was deemed to be rationale from a prognostic standpoint, the data regarding the prognostic significance of para-aortic lymph node metastasis were still limited, especially for endometrioid type with a favorable prognosis. The study by McMeekin et al. (18) demonstrated that patients with positive para-aortic lymph node had more extensive disease, but no significant survival difference compared with those with disease confined to the pelvic lymph node. Nevertheless, other studies also proposed that para-aortic lymph node metastasis confers a lower overall survival and disease-free survival (19). A recent study based on the SEER database showed that endometrioid tumors with stage IIIC2 were associated with higher all-cause and CSM compared with those with IIIC1 stage (20). However, this study incorporated patients with non-endometrioid histologic type, such as serious and clear cells, which were demonstrated to be more common among stage IIIC2 and had a significantly worse prognosis than those with endometrioid type. At odds with the results of this study, our multivariate results based on competing risk model confirmed that patients of endometrioid type who had aortic lymph node metastasis (IIIC2) did not experience significantly higher CSM than those with pelvic lymph node metastasis (IIIC1) alone. Moreover, our data further confirmed that aortic lymph node metastasis conferred no prognostic value, especially in subgroups of IIIC patients stratified by T stage. Our data, to some extent, implied that it is of little significance to further divide IIIC stage into IIIC1 and IIIC2, at least in patients of endometrioid type.

Given that the current FIGO staging system assigned all patients with positive lymph nodes to IIIC stage without regard to the extent of extrauterine invasion, it seems reasonable to postulate that tumors with severe extrauterine invasion represent more aggressive or more advanced disease. It was reported that patients who had both adnexal invasion and positive pelvic lymph nodes experience worse outcomes in comparison with patients who had pelvic lymph node metastases alone (21). Other studies also observed that the number of extrauterine sites, including serosa, adnexa, vagina, or parametrium, exerts as strong prognostic factors in III endometrial cancer (22–24). Indeed, our data highlighted that the T stage was significantly associated with the prognosis of IIIC patients. Although regional lymph node metastasis has occurred in all patients, the prognosis of patients with T3 stage was significantly worse than that of patients with T1 stage (HR = 2.64, *p* < 0.001). Therefore, the FIGO IIIC substage, which is based on the involvement of regional lymph node, is sometimes not satisfactory due to inconsistency with the original purpose of clinical staging, that is, to better distinguish the severity or prognosis of the disease.

Recently, sentinel lymph node mapping has been advocated as an alternative staging technique for endometrial cancer (25–27). Rossi et al. (28) demonstrated that the sentinel lymph node technique has a good diagnostic accuracy in detecting regional lymph node metastases, which can be used safely to replace lymphadenectomy in the staging of endometrial cancer (29). Meanwhile, the majority of patients who had received sentinel lymph dissection biopsy did not assess the para-aortic lymph node metastasis due to the low incidence of isolated aortic lymph node metastasis (30). Also, because complete lymphadenectomy is associated with major comorbidities, including lymphoedema, genitofemoral nerve injury, and lymphocyst formation, sentinel lymph dissection seems to be an appropriate management strategy to reduce surgical complications (31, 32). Furthermore, two large prospective clinical trials failed to demonstrate the survival benefit of the addition of pelvic lymphadenectomy compared with that of standard hysterectomy and bilateral salpingo-oophorectomy alone (15, 33). Therefore, it seems to be less meaningful to perform a thorough surgical staging for IIIC endometrial cancer patients by complete pelvic and para-aortic lymphadenectomy. Further optimization of the FIGO IIIC staging to distinguish patients with distinct prognosis better is of increasing clinical significance for personalized management.

Due to the relatively poor prognostic value of FIGO IIIC staging, especially in patients with endometrioid type, we established a new IIIC subdivision system and divided patients with T1N1 or T1N2 into IIICa due to the relatively similar prognosis. Those with T2N1 or T2N2 were categorized as IIICb, whereas the rest of the IIIC patients with T3N1 or T3N2 were staged as IIICc. Interestingly, based on multivariate competing risk analysis, our findings suggested that patients with different T stages had dramatically different survival outcomes, whereas N stage made no sense in the patient's prognosis. In our study, patients with IIICb disease had an ~50% increased mortality risk, and those with IIICc disease showed more than 2.5-fold higher risk of mortality compared with those with IIICa disease. Similar trends of the prognostic difference were observed across the validation and total sets.

The results of our study might have some implications for clinical practice. Although endometrial cancer with stage III has been demonstrated as a heterogeneous disease with distinct subtypes (11), the subsequent management of substages IIIC1 and IIIC2 are often treated similarly. It is reasonable to hypothesize that substages, which represent distinct and separate entities, should be treated with optimized and personalized adjuvant therapy based on prognosis. Recently, practice pattern remains subject of debate about the adjuvant management of patients with stage IIIC endometrial cancer. Three previously published randomized trials compared adjuvant chemotherapy with radiotherapy and found no difference in overall survival and progression-free survival (34–36). Since pelvic relapse has been reported frequently in patients who had undergone chemotherapy alone (37, 38), the combination of radiotherapy and chemotherapy was proposed to maximize both local and distant control (39, 40). The updated analysis of the Postoperative Radiation Therapy in Endometrial Carcinoma 3 has exhibited significantly improved overall survival and failure-free survival in stage III patients treated with chemoradiotherapy vs. radiotherapy alone (41). On the contrary, results from Gynecologic Oncology Group 258 failed to demonstrate a significant benefit for combined treatment modality compared with chemotherapy alone in locally advanced endometrial cancer (42). Due to the controversial results for the optimal postoperative approach to locoregionally advanced endometrial cancer, multiple comparisons of the survival benefit between various combined treatment modalities in our new subcategories for IIIC patients were conducted in our study. Our study presented that postoperative adjuvant chemotherapy or radiotherapy alone did not improve survival for patients categorized as IIICa or IIICb, and the combination of chemotherapy and chemotherapy should be considered due to the survival gain observed for those two subcategories. For patients with IIICc disease, all of the combined treatment modalities could improve survival, whereas the combination of chemotherapy and radiotherapy was preferred due to the ~55% reduction in mortality risk. Overall, our study demonstrated that combination therapy with postoperative chemotherapy and radiotherapy exerted as the optimal choice for endometrial cancer patients with IIIC stage. However, considering the existence of selection bias, we believe that further investigation is required.

Considering the dramatically prognostic variance for IIIC endometrial cancer patients, a nomogram capable of predicting the long-term CSM would be useful to inform clinical decision-making (43). Hence, a nomogram that incorporated independent prognostic indicators in multivariate competing risk model was constructed. Our nomogram exhibited good predictive capabilities with a C-index above 0.700 both in the training and validation sets, which serve as good as several widely accepted nomograms (44–46).

Inevitably, this work has several limitations. Firstly, some variables were not available or accessible in the SEER program, such as menopausal status, body mass index, peritoneal cytology, and lymphovascular space invasion, which were reported to affect the prognosis of endometrial cancer (4). Secondly, details of adjuvant therapy in terms of radiation treatment field design, radiation dose, fractionation, elapsed days, and pharmaceutical information of chemotherapy are currently not documented in SEER. It should be noted that inappropriately administered adjuvant therapy may have a significant impact on overall treatment outcomes. Thirdly, considering the small sample size of the subgroups receiving chemotherapy only, radiotherapy only, or pure chemoradiotherapy, the exact survival benefit of those therapeutic options should be compared with other treatment modalities in well-designed prospective clinical trials with large sample size. Fourthly, our study is limited by the intrinsic weaknesses of retrospective databases, wherein selection bias is inherent.

## Conclusions

This study suggests no prognostic difference between FIGO IIIC1 and IIIC2 patients with endometrioid-type endometrial cancer. A new subdivision of IIIC category was reported herein, possessing a good ability to distinguish IIIC stage patients with a distinct prognosis. Although personalized treatment choice needs to be considered, combination therapy of postoperative chemotherapy and radiotherapy exerted as the optimal choice for endometrioid cancer patients with IIIC stage. Also, a nomogram based on a competing risk model was constructed, which may assist clinicians in assessing each IIIC patient's prognosis better.

## Data Availability Statement

Publicly available datasets were analyzed in this study. This data can be found here: https://seer.cancer.gov/.

## Author Contributions

LW and GZ contributed to the idea and design. LW, GZ, JW, YZ, and SC contributed to the data acquisition and analysis. LW, WH, and GZ contributed to the manuscript writing and revision. All authors have read and approved the final version of this manuscript.

## Conflict of Interest

The authors declare that the research was conducted in the absence of any commercial or financial relationships that could be construed as a potential conflict of interest.
