# Supplementary material for: Subdivision of IIIC Stage for Endometrioid Carcinoma to Better Predict Prognosis and Treatment Guidance
Source: Front Oncol. 2020 Jul 31;10:1175. doi: 10.3389/fonc.2020.01175 (PMC7411261; doi:10.3389/fonc.2020.01175)
Supplement: Supplementary file 1 [file Table_1.DOCX]

Table S1. Baseline Patient Characteristics (N = 3591)

|  | Training Set  n (%) | Validation Set  n (%) | P value |
| --- | --- | --- | --- |
| Year of diagnosis |  |  | 0.987 |
| 2004-2006 | 327 (18.2) | 333 (18.5) |  |
| 2007-2009 | 388 (21.6) | 386 (21.5) |  |
| 2010-2012 | 506 (28.2) | 511 (28.5) |  |
| 2013-2015 | 574 (32.0) | 566 (31.5) |  |
| Age, y |  |  | 0.386 |
| <41 | 46 (2.6) | 57 (3.2) |  |
| 41-60 | 818 (45.6) | 850 (47.3) |  |
| 61-80 | 834 (46.5) | 803 (44.7) |  |
| >80 | 97 (5.4) | 86 (4.8) |  |
| Race |  |  | 0.125 |
| Black | 171 (9.5) | 141 (7.9) |  |
| White | 1423 (79.3) | 1431 (79.7) |  |
| Other | 201 (11.2) | 224 (12.5) |  |
| Marital status |  |  | 0.660 |
| Unmarried | 875 (48.7) | 882 (49.1) |  |
| Married | 852 (47.5) | 856 (47.7) |  |
| Unknown | 68 (3.8) | 58 (3.2) |  |
| Histologic grade |  |  | 0.518 |
| Well differentiated | 324 (18.1) | 305 (17.0) |  |
| Moderately differentiated | 600 (33.4) | 646 (36.0) |  |
| Poorly differentiated | 478 (26.6) | 464 (25.8) |  |
| Undifferentiated | 71 (4.0) | 77 (4.3) |  |
| Unknown | 322 (17.9) | 304 (16.9) |  |
| FIGO stage |  |  | 0.209 |
| IIIC1 | 1190 (66.3) | 1226 (68.3) |  |
| IIIC2 | 605 (33.7) | 570 (31.7) |  |
| T category |  |  | 0.891 |
| T1 | 910 (50.7) | 907 (50.5) |  |
| T2 | 363 (20.2) | 355 (19.8) |  |
| T3 | 522 (29.1) | 534 (29.7) |  |
| Treatment modality |  |  | 0.124 |
| None | 11 (0.6) | 14 (0.8) |  |
| Surgery only | 289 (16.1) | 310 (17.3) |  |
| Chemotherapy only | 6 (0.3) | 16 (0.9) |  |
| Radiation only | 12 (0.7) | 11 (0.6) |  |
| Surgery + Chemo | 384 (21.4) | 409 (22.8) |  |
| Surgery + Rad | 244 (13.6) | 243 (13.5) |  |
| Chemo + Rad | 19 (1.1) | 9 (0.5) |  |
| Surgery + Chemo + Rad | 830 (46.2) | 784 (43.7) |  |

Abbreviations: Chemo, Chemotherapy; Rad, Radiation.
