# Supplementary material for: Subdivision of IIIC Stage for Endometrioid Carcinoma to Better Predict Prognosis and Treatment Guidance
Source: Front Oncol. 2020 Jul 31;10:1175. doi: 10.3389/fonc.2020.01175 (PMC7411261; doi:10.3389/fonc.2020.01175)
Supplement: Supplementary file 2 [file Table_2.DOCX]

Table S2. Association of Cancer-specific Mortality with different IIIc subgroup.

|  | Model 1 | |  | Model 2 | |  | Model 3 | |  | Model 4 | |  | Model 5 | |  |
| --- | --- | --- | --- | --- | --- | --- | --- | --- | --- | --- | --- | --- | --- | --- | --- |
|  | SHR(95% CI) | P Value |  | SHR(95% CI) | P Value |  | SHR(95% CI) | P Value |  | SHR(95% CI) | P Value |  | SHR(95% CI) | P Value |  |
| Part I: univariate analysis | | | | | | | | | | | | | | | |
| T1N1 | Ref |  |  |  |  |  |  |  |  |  |  |  |  |  |  |
| T1N2 | 1.06 (0.75-1.51) | 0.725 |  | Ref |  |  |  |  |  |  |  |  |  |  |  |
| T2N1 | 1.72 (1.28-2.32) | <0.001 |  | 1.62 (1.11-2.35) | 0.011 |  | Ref |  |  |  |  |  |  |  |  |
| T2N2 | 1.86 (1.25-2.76) | 0.002 |  | 1.74 (1.10-2.75) | 0.017 |  | 1.08 (0.71-1.64) | 0.733 |  | Ref |  |  |  |  |  |
| T3N1 | 3.31 (2.58-4.26) | <0.001 |  | 3.08 (2.20-4.31) | <0.001 |  | 1.91 (1.44-2.54) | <0.001 |  | 1.77 (1.20-2.62) | 0.004 |  | Ref |  |  |
| T3N2 | 3.50 (2.66-4.61) | <0.001 |  | 3.26 (2.29-4.65) | <0.001 |  | 2.02 (1.49-2.75) | <0.001 |  | 1.88 (1.25-2.82) | 0.002 |  | 1.06 (0.81-1.37) | 0.679 |  |
| Part II: multivariate analysis^a^ | | | | | | | | | | | | |  |  |  |
| T1N1 | Ref |  |  |  |  |  |  |  |  |  |  |  |  |  |  |
| T1N2 | 1.06 (0.75-1.50) | 0.759 |  | Ref |  |  |  |  |  |  |  |  |  |  |  |
| T2N1 | 1.54 (1.14-2.08) | 0.005 |  | 1.50 (1.03-2.19) | 0.036 |  | Ref |  |  |  |  |  |  |  |  |
| T2N2 | 1.74 (1.16-2.60) | 0.007 |  | 1.65 (1.04-2.61) | 0.032 |  | 1.09 (0.71-1.67) | 0.701 |  | Ref |  |  |  |  |  |
| T3N1 | 2.92 (2.26-3.77) | <0.001 |  | 2.80 (1.99-3.93) | <0.001 |  | 1.87 (1.40-2.51) | <0.001 |  | 1.70 (1.15-2.53) | 0.008 |  | Ref |  |  |
| T3N2 | 2.75 (2.07-3.65) | <0.001 |  | 2.61 (1.82-3.75) | <0.001 |  | 1.76 (1.28-2.41) | 0.001 |  | 1.60 (1.05-2.42) | 0.027 |  | 1.71 (0.63-4.68) | 0.581 |  |

Abbreviation: SHR, subdistribution hazard ratio

^a^ The multivariate analysis model was adjusted for age, race, marital status, histologic grade, and treatment (surgery, chemotherapy, and radiation).
