# Supplementary material for: Subdivision of IIIC Stage for Endometrioid Carcinoma to Better Predict Prognosis and Treatment Guidance
Source: Front Oncol. 2020 Jul 31;10:1175. doi: 10.3389/fonc.2020.01175 (PMC7411261; doi:10.3389/fonc.2020.01175)
Supplement: Supplementary file 3 [file Table_3.docx]

Table S3. Multivariate analysis of cancer-specific mortality: A competing risk model^a^

|  | Training Set | |  | Validation Set | |  | Whole Set | |
| --- | --- | --- | --- | --- | --- | --- | --- | --- |
|  | SHR (95% CI) | P value |  | SHR (95% CI) | P value |  | SHR (95% CI) | P value |
| IIIC subdivision |  |  |  |  |  |  |  |  |
| IIICa | Ref |  |  | Ref |  |  | Ref |  |
| IIICb | 1.53 (1.18-1.98) | 0.001 |  | 1.35 (1.05-1.74) | 0.021 |  | 1.45 (1.21-1.73) | <0.001 |
| IIICc | 2.64 (2.13-3.28) | <0.001 |  | 2.45 (1.98-3.03) | <0.001 |  | 2.52 (2.17-2.93) | <0.001 |
| Age, y |  |  |  |  |  |  |  |  |
| <40 | Ref |  |  | Ref |  |  | Ref |  |
| 41-60 | 1.14 (0.62-2.12) | 0.660 |  | 1.17 (0.61-2.27) | 0.640 |  | 1.13 (0.73-1.77) | 0.580 |
| 61-80 | 1.89 (1.03-3.47) | 0.041 |  | 1.46 (0.76-2.82) | 0.260 |  | 1.60 (1.03-2.49) | 0.036 |
| >81 | 2.90 (1.50-5.63) | 0.002 |  | 2.66 (1.27-5.55) | 0.009 |  | 2.66 (1.63-4.33) | <0.001 |
| Race |  |  |  |  |  |  |  |  |
| Black | Ref |  |  | Ref |  |  | Ref |  |
| White | 0.73 (0.55-0.97) | 0.028 |  | 0.60 (0.46-0.79) | <0.001 |  | 0.69 (0.56-0.85) | <0.001 |
| Other | 0.65 (0.44-0.96) | 0.030 |  | 0.59 (0.40-0.79) | 0.007 |  | 0.66 (0.50-0.87) | 0.005 |
| Marital status |  |  |  |  |  |  |  |  |
| Unmarried | Ref |  |  | Ref |  |  | Ref |  |
| Married | 0.77 (0.64-0.94) | 0.009 |  | 0.98 (0.81-1.19) | 0.860 |  | 0.88 (0.77-1.01) | 0.067 |
| Unknown | 0.67 (0.35-1.28) | 0.220 |  | 1.49 (0.96-2.29) | 0.07 |  | 1.05 (0.72-1.54) | 0.810 |
| Histologic grade |  |  |  |  |  |  |  |  |
| Grade 1 | Ref |  |  | Ref |  |  | Ref |  |
| Grade 2 | 1.42 (1.02-1.98) | 0.040 |  | 1.06 (0.77-1.46) | 0.700 |  | 1.23 (0.98-1.55) | 0.077 |
| Grade 3 | 2.51 (1.79-3.51) | <0.001 |  | 2.38 (1.76-3.21) | <0.001 |  | 2.44 (1.95-3.06) | <0.001 |
| Grade 4 | 3.35 (2.13-5.26) | <0.001 |  | 3.04 (1.94-4.75) | <0.001 |  | 3.14 (2.27-4.33) | <0.001 |
| Unknown | 2.04 (1.41-1.98) | <0.001 |  | 1.24 (0.86-1.80) | 0.250 |  | 1.60 (1.23-2.07) | <0.001 |
| FIGO stage |  |  |  |  |  |  |  |  |
| IIIC1 | Ref |  |  | Ref |  |  | Ref |  |
| IIIC2 | 1.03 (0.85-1.26) | 0.760 |  | 1.19 (0.99-1.44) | 0.069 |  | 1.11 (0.97-1.28) | 0.13 |
| Surgery |  |  |  |  |  |  |  |  |
| No | Ref |  |  | Ref |  |  | Ref |  |
| Yes | 0.17 (0.11-0.26) | <0.001 |  | 0.19 (0.12-0.28) | <0.001 |  | 0.20 (0.15-0.27) | <0.001 |
| Chemotherapy |  |  |  |  |  |  |  |  |
| No | Ref |  |  | Ref |  |  | Ref |  |
| Yes | 0.70 (0.57-0.85) | <0.001 |  | 0.79 (0.65-0.96) | 0.019 |  | 0.76 (0.66-0.88) | <0.001 |
| Radiotherapy |  |  |  |  |  |  |  |  |
| No | Ref |  |  | Ref |  |  | Ref |  |
| Yes | 0.74 (0.61-0.88) | 0.001 |  | 0.56 (0.47-0.67) | <0.001 |  | 0.70 (0.61-0.80) | <0.001 |

Abbreviations: SHR, subdistribution hazard ratio

^a^Adjusted variables included age, race, marital status, histologic grade, FIGO stage, and treatment including surgery, chemotherapy, and radiation.
