# Supplementary material for: Subdivision of IIIC Stage for Endometrioid Carcinoma to Better Predict Prognosis and Treatment Guidance
Source: Front Oncol. 2020 Jul 31;10:1175. doi: 10.3389/fonc.2020.01175 (PMC7411261; doi:10.3389/fonc.2020.01175)
Supplement: Supplementary file 4 [file Table_4.docx]

Table S4. Multivariate analysis of all-cause mortality: Cox proportional hazards model^a^

|  | Training Set | |  | Validation Set | |  | Whole Set | |
| --- | --- | --- | --- | --- | --- | --- | --- | --- |
|  | HR (95% CI) | P value |  | HR (95% CI) | P value |  | HR (95% CI) | P value |
| IIIC subdivision |  |  |  |  |  |  |  |  |
| IIICa | Ref |  |  | Ref |  |  | Ref |  |
| IIICb | 1.51 (1.21-1.90) | <0.001 |  | 0.33 (1.02-1.58) | 0.033 |  | 1.41 (1.21-1.65) | <0.001 |
| IIICc | 2.67 (2.21-3.23) | <0.001 |  | 2.27 (1.88-2.73) | <0.001 |  | 2.45 (2.15-2.80) | <0.001 |
| Age, y |  |  |  |  |  |  |  |  |
| <40 | Ref |  |  | Ref |  |  | Ref |  |
| 41-60 | 1.36 (0.76-2.44) | 0.302 |  | 0.99 (0.57-1.74) | 0.980 |  | 1.18 (0.79-1.77) | 0.428 |
| 61-80 | 2.61 (1.46-4.65) | 0.001 |  | 1.51 (0.86-2.63) | 0.260 |  | 2.01 (1.34-3.00) | 0.001 |
| >81 | 5.22 (2.80-9.72) | <0.001 |  | 3.23 (1.76-5.92) | <0.001 |  | 4.06 (2.63-6.25) | <0.001 |
| Race |  |  |  |  |  |  |  |  |
| Black | Ref |  |  | Ref |  |  | Ref |  |
| White | 0.75 (0.58-0.98) | 0.035 |  | 0.64 (0.50-0.81) | <0.001 |  | 0.70 (0.59-0.84) | <0.001 |
| Other | 0.64 (0.44-0.92) | 0.016 |  | 0.60 (0.42-0.85) | 0.004 |  | 0.64 (0.49-0.82) | <0.001 |
| Marital status |  |  |  |  |  |  |  |  |
| Unmarried | Ref |  |  | Ref |  |  | Ref |  |
| Married | 0.73 (0.61-0.86) | <0.001 |  | 0.93 (0.78-1.10) | 0.370 |  | 0.83 (0.74-0.94) | 0.002 |
| Unknown | 0.69 (0.40-1.19) | 0.179 |  | 1.27 (0.84-1.93) | 0.264 |  | 0.95 (0.68-1.32) | 0.761 |
| Histologic grade |  |  |  |  |  |  |  |  |
| Grade 1 | Ref |  |  | Ref |  |  | Ref |  |
| Grade 2 | 1.52 (1.13-2.04) | 0.006 |  | 1.19 (0.91-1.56) | 0.204 |  | 1.32 (1.08-1.60) | 0.006 |
| Grade 3 | 2.45 (1.83-3.29) | <0.001 |  | 2.24 (1.72-2.92) | <0.001 |  | 2.30 (1.90-2.80) | <0.001 |
| Grade 4 | 3.90 (2.61-5.84) | <0.001 |  | 2.73 (1.85-4.04) | <0.001 |  | 3.13 (2.37-4.13) | <0.001 |
| Unknown | 1.76 (1.25-2.47) | <0.001 |  | 1.23 (0.89-1.70) | 0.215 |  | 1.45 (1.15-1.82) | 0.002 |
| FIGO stage |  |  |  |  |  |  |  |  |
| IIIC1 | Ref |  |  | Ref |  |  | Ref |  |
| IIIC2 | 1.01 (0.85-1.20) | 0.891 |  | 1.27 (1.02-1.58) | 0.033 |  | 1.11 (0.99-1.25) | 0.075 |
| Surgery |  |  |  |  |  |  |  |  |
| No | Ref |  |  | Ref |  |  | Ref |  |
| Yes | 0.21 (0.15-0.29) | <0.001 |  | 0.15 (0.11-0.21) | <0.001 |  | 0.20 (0.15-0.25) | <0.001 |
| Chemotherapy |  |  |  |  |  |  |  |  |
| No | Ref |  |  | Ref |  |  | Ref |  |
| Yes | 0.67 (0.56-0.79) | <0.001 |  | 0.75 (0.63-0.89) | 0.001 |  | 0.71 (0.63-0.80) | <0.001 |
| Radiotherapy |  |  |  |  |  |  |  |  |
| No | Ref |  |  | Ref |  |  | Ref |  |
| Yes | 0.80 (0.68-0.95) | 0.010 |  | 0.60 (0.51-0.71) | <0.001 |  | 0.70 (0.62-0.78) | <0.001 |

Abbreviations: HR, hazard ratio.

^a^Adjusted variables included age, race, marital status, histologic grade, FIGO stage, and treatment including surgery, chemotherapy, and radiation.
