# Supplementary material for: Subdivision of IIIC Stage for Endometrioid Carcinoma to Better Predict Prognosis and Treatment Guidance
Source: Front Oncol. 2020 Jul 31;10:1175. doi: 10.3389/fonc.2020.01175 (PMC7411261; doi:10.3389/fonc.2020.01175)
Supplement: Supplementary file 5 [file Table_5.DOCX]

Table S5. Patient demographics of various treatment modalities (N = 3493)

|  | Sugery only  n (%) | Surgery+chemo  n (%) | Surgery+rad  n (%) | Surgery+chemo+rad  n (%) | P value |
| --- | --- | --- | --- | --- | --- |
| Year of diagnosis |  |  |  |  | <0.001 |
| 2004-2006 | 149 (24.9) | 81 (10.2) | 212 (43.5) | 209 (12.9) |  |
| 2007-2009 | 148 (24.7) | 185 (23.3) | 113 (23.2) | 316 (19.6) |  |
| 2010-2012 | 160 (26.7) | 238 (30.0) | 98 (20.1) | 491 (30.4) |  |
| 2013-2015 | 142 (23.7) | 289 (36.4) | 64 (13.1) | 598 (37.1) |  |
| Age, y |  |  |  |  | <0.001 |
| <41 | 10 (1.7) | 32 (4.0) | 14 (2.9) | 44 (2.7) |  |
| 41-60 | 232 (38.7) | 377 (47.5) | 180 (37.0) | 845 (52.4) |  |
| 61-80 | 286 (47.7) | 356 (44.9) | 244 (50.1) | 701 (43.4) |  |
| >80 | 71 (11.9) | 28 (3.5) | 49 (10.1) | 24 (1.5) |  |
| Race |  |  |  |  | 0.001 |
| Black | 52 (8.7) | 80 (10.1) | 50 (10.3) | 117 (7.2) |  |
| White | 474 (79.1) | 590 (74.4) | 389 (79.9) | 1322 (81.9) |  |
| Other | 73 (12.2) | 123 (15.5) | 48 (9.9) | 175 (10.8) |  |
| Marital status |  |  |  |  | <0.001 |
| Unmarried | 329 (54.9) | 401 (50.6) | 246 (50.5) | 715 (44.3) |  |
| Married | 248 (41.4) | 367 (46.3) | 225 (46.2) | 840 (52.0) |  |
| Unknown | 22 (3.7) | 25 (3.2) | 16 (3.3) | 59 (3.7) |  |
| Histologic grade |  |  |  |  | <0.001 |
| Grade 1 | 105 (17.5) | 139 (17.5) | 100 (20.5) | 268 (16.6) |  |
| Grade 2 | 200 (33.4) | 234 (29.5) | 177 (36.3) | 607 (37.6) |  |
| Grade 3 | 180 (30.1) | 249 (31.4) | 140 (28.7) | 345 (21.4) |  |
| Grade 4 | 18 (3.0) | 47 (5.9) | 18 (3.7) | 61 (3.8) |  |
| Unknown | 96 (16.0) | 124 (15.6) | 52 (10.7) | 333 (20.6) |  |
| FIGO stage |  |  |  |  | <0.001 |
| IIIC1 | 413 (68.9) | 490 (61.8) | 363 (74.5) | 1091 (67.6) |  |
| IIIC2 | 186 (31.1) | 303 (38.2) | 124 (25.5) | 523 (32.4) |  |
| T category |  |  |  |  | <0.001 |
| T1 | 297 (49.6) | 393 (49.6) | 263 (54.0) | 846 (52.4) |  |
| T2 | 131 (21.9) | 127 (16.0) | 116 (23.8) | 324 (20.1) |  |
| T3 | 171 (28.5) | 273 (34.4) | 108 (22.2) | 444 (27.5) |  |

Abbreviations: Chemo, Chemotherapy; Rad, Radiation.
