# Supplementary figures and images for: Subdivision of IIIC Stage for Endometrioid Carcinoma to Better Predict Prognosis and Treatment Guidance
Source: Front Oncol. 2020 Jul 31;10:1175. doi: 10.3389/fonc.2020.01175 (PMC7411261; doi:10.3389/fonc.2020.01175)

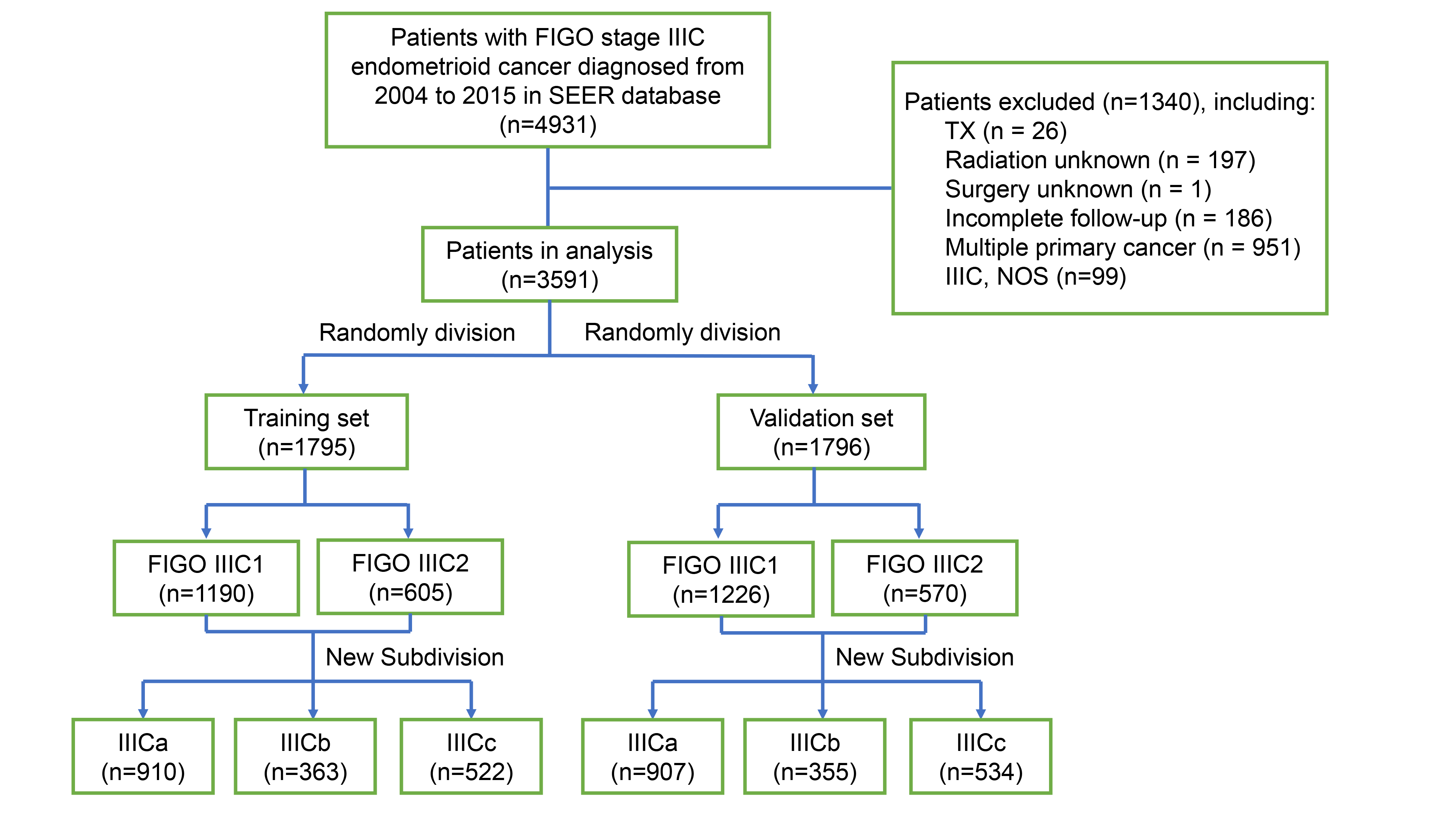

Supplement: Supplementary file 6 [file Image_1.tif]

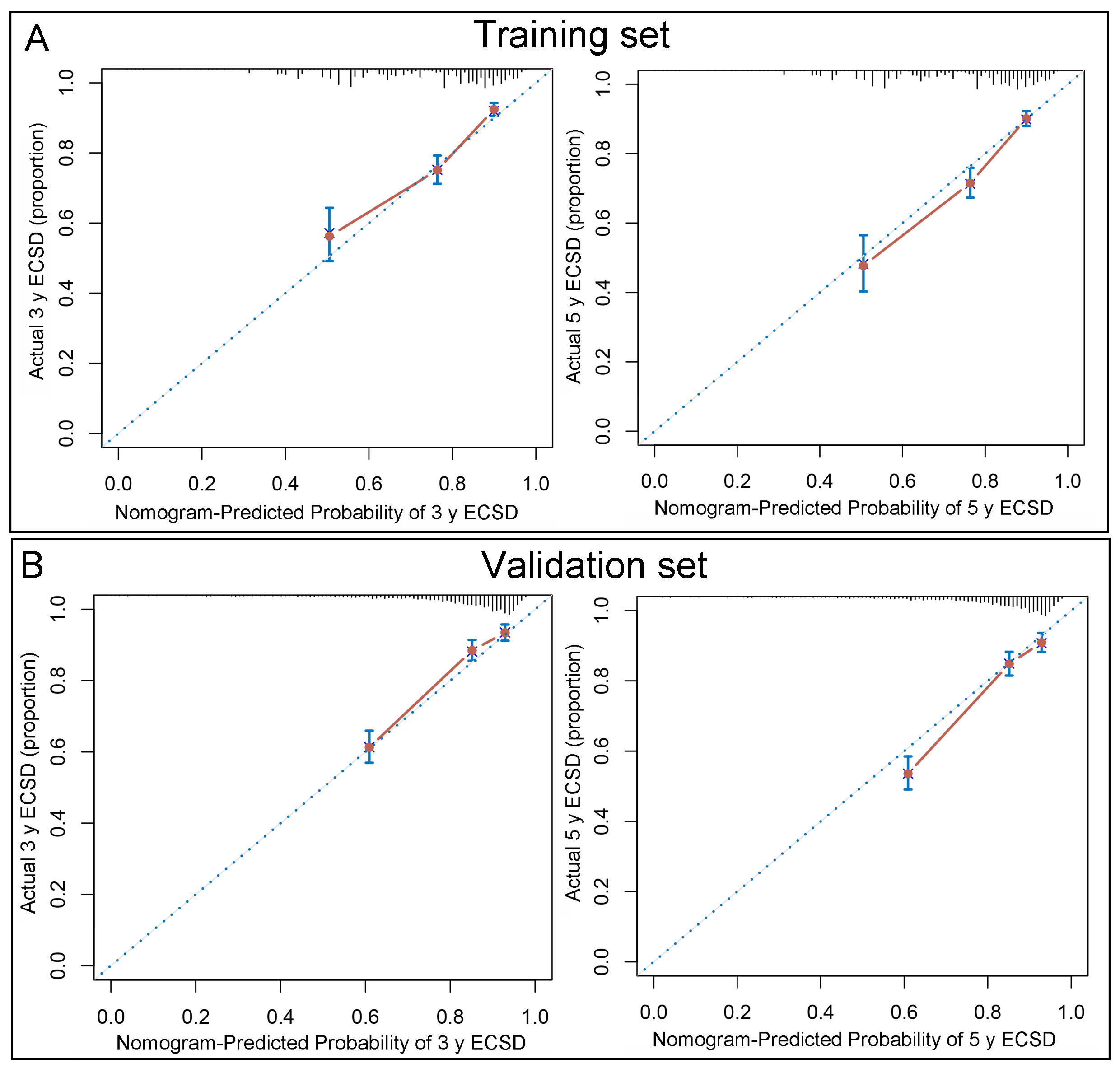

Supplement: Supplementary file 7 [file Image_2.tif]
